# Developmental kindergarten classroom intervention for spatial relational terms

**DOI:** 10.1590/2317-1782/20212021176

**Published:** 2022-05-09

**Authors:** Maggie Eising, Courtney Karasinski

**Affiliations:** 1 Department of Communication Sciences and Disorders, Grand Valley State University – GVSU - Grand Rapids (MI), United States of America.

**Keywords:** Relational Terms, Spatial Terms, Service-Delivery Models, Response to Intervention, Multi-Tiered System of Support

## Abstract

**Purpose:**

Relational ability is a key attribute of language. Knowledge of relational terms, including spatial terms, can facilitate development of relational ability. Acquisition of spatial terms can be challenging and necessitates experience and input due to the abstractness of the concepts. Service delivery models for school-based speech-language pathologists (SLPs) are changing from traditional “pull-out” therapy to intervention in the classroom. Response to Intervention (RtI) and multi-tiered system of support (MTSS) frameworks have expanded SLPs’ roles to working with all children at-risk for academic difficulties.

**Methods:**

Given the importance of spatial terms, and the changing roles and service delivery models for school-based SLPs, this investigation evaluated a six-week classroom-based intervention targeting spatial terms in a developmental kindergarten classroom of five-year-old children.

**Results:**

At post-test, more than half of the children who did not understand the targeted spatial terms at pre-test demonstrated understanding of the words first, front, last, behind, center, below, under, and right by correctly identifying pictures representing these words. Around and left were the only two words learned by fewer than half of the children.

**Conclusion:**

These findings augment research used by SLPs providing language support to children within the first tier of Response to Intervention or multi-tiered system of support.

## INTRODUCTION

Relational ability has been postulated as a key attribute that differentiates humans from other species^(1,2)^. The use of relational ability, also termed *relational cognition*, is essential and pervasive in everyday conversations and in academic contexts. Language influences the development of relational cognition, from both developmental and language evolutionary perspectives^(3)^.

Relational terms can be difficult for children to acquire. Whereas entity terms, often denoted by nouns, are observable phenomena in the world; relational terms, often verbs and prepositions, convey *connections* among things in the world. These terms are more abstract and less observable. Verbs and prepositions are often more difficult to understand and utilize than nouns, and they have more meanings that vary across contexts. In order to understand these connections and the terms used to describe them, young children require experience and input from adults^(4)^. The current investigation focused on one category of relational terms: spatial terms.

A number of studies have demonstrated that the comprehension and production of spatial terms are critical for performing spatial cognition tasks. Young children whose parents use many spatial terms in conversation are likely to use more spatial language and perform better on spatial tasks, including spatial transformations, block design, and spatial analogies^(5)^. Preschoolers who know *left* and *right* more effectively use landmarks to find hidden objects than peers who have not acquired these terms^(6)^. Those who know *middle* exhibit better performance on a midpoint search task than those who do not understand *middle* ^(7)^. Children perform better on spatial cognition tasks when examiners use particular spatial terms, including *top*, *bottom*, *on*, *in*, *under* ^(8)^, *middle* ^(8,9)^, *left,* and *right* ^(10)^.

Miller et al.^(11)^ considered adaptive use of language in relation to the role of language in spatial cognition. They revealed that, in four-year-old children, quantity of spatial terms produced significantly predicted performance on spatial tasks, beyond the variance accounted for by the demographic factors of age and gender. When adaptive use of spatial terms was added to the model, it was a significant predictor beyond all of the other factors, and quantity of spatial terms was no longer significant. These findings suggest that mere production of spatial terms is not sufficient to facilitate performance on spatial tasks, but that children need to use their knowledge of spatial terms in a manner that is relevant to the task. This relation between use of spatial terms in context and performance on spatial tasks highlights the need to learn these words in meaningful contexts.

In the United States, the Common Core State Standards (CCSS) have been adopted as the learning goals for children in kindergarten (typically beginning at age 5) through high school (typically ending at age 18) by a majority of states for mathematics and English language arts, and the Next Generation Science Standards^(12)^ have been adopted by a majority of states for science. In the CCSS for Mathematics^(13)^ spatial relations are specifically highlighted as one of the two areas of focus for kindergarten mathematics. The CCSS for English Language Arts^(14)^ specify the use of the most frequently used prepositions in standard 1e for kindergarten students, and the list of examples includes the spatial terms *in*, *out*, *on*, *off*, and *by*. Spatial relations are specifically mentioned in the NGSS earth science standards for middle school and high school. Although spatial terms are not specifically mentioned in the standards for younger students or in the physical or life sciences standards, they would need to be understood in order to carry out a variety of directions in many of the activities used to teach science, such as conducting experiments.

In addition to the impact on academic pursuits, the understanding and use of spatial terms affects social communication. Increasingly, communication takes place outside of face-to-face contexts, such as via phone conversations, text messages, and social media postings. Due to the ambiguity of spatial terms, especially in contexts that are not face-to-face, their use can lead to miscommunications when communication partners do not have a shared understanding^(15)^.

### Service delivery models

The role of the school-based speech-language pathologist (SLP) is changing. In the past, many school-based SLPs provided mostly “pull-out” services, in which children worked individually or in small groups with the SLP in a space separate from their general classroom. Current practices are moving away from these traditional pull-out services toward serving children in the classroom setting in collaboration with classroom teachers. Educational legislation and initiatives in the United States support classroom-based intervention models. The Individuals with Disabilities Education Improvement Act of 2004^(16)^ highlights the importance of educating students in the *least restrictive environment (LRE)*, which, for many students with deficits in language, may be the general education classroom. The English Language Arts and Literacy CCSS highlight the relations among reading, writing, speaking, and listening and advocate for an interdisciplinary approach to teaching these skills. SLPs are recognizing their crucial role in facilitating language development in all students who are at risk for academic challenges, as recommended by the American Speech-Language-Hearing Association^(17)^. Thus, many schools now provide speech-language services within a *Response to Intervention* (RtI) or *multi-tiered systems of support* (MTSS) framework. This service delivery model benefits all students who need additional help, whether or not they qualify for services under a disability category. Typically, instruction is provided within three tiers. Tier 1 provides evidence-based instruction for all students, and progress is monitored using curriculum-based assessments. Tier 2 instruction is provided to students who do not meet the progress expectations^(18)^. This level of specialized instruction is often provided in a small group^(19)^. Tier 3 instruction is provided to students who continue to exhibit difficulty, even with Tier 2 instruction. Tier 3 typically includes a referral for a comprehensive special education evaluation^(18)^.

Despite an increasing awareness of the SLP’s role in providing support for oral and written language acquisition for all students, few studies have addressed the efficacy of classroom-based services. Cirrin et al.’s systematic review assessing the impact of service delivery model on communication outcomes for elementary school children revealed a dearth of evidence on this topic and highlighted the need for further research^(20)^. The few studies that have been conducted on providing language intervention in the classroom have indicated positive effects of the intervention. Throneburg, Calvert, Sturm, Paramboukas, and Paul compared the effect on vocabulary skills of a collaborative approach between the SLP and classroom teacher, a classroom-based intervention delivered by the SLP, and a traditional pull-out model for kindergarten through third grade children qualifying for speech-language services. These approaches were compared for the students in the classrooms who did not qualify for speech-language services. Both the collaborative model and the SLP providing classroom-based instruction resulted in gains in vocabulary skills for the children with typical speech-language development, and the collaborative model yielded the best outcomes for the children who qualified for speech-language services^(21)^. Gillam, Olswzeski, Fargo, and Gillam revealed that children receiving narrative and vocabulary instruction provided by an SLP in the classroom made greater gains on narrative and vocabulary measures than the children a comparison classroom who did not receive the intervention. Within the classroom receiving the intervention, the children with high risk for language impairment made greater gains in narrative ability and fewer gains in vocabulary than the children at low risk for language impairment^(22)^. Similarly, Lennox, Westerveld, and Trembath provided a classroom-based intervention to address emergent literacy skills in children during their first year of formal education (age 5 years). The children in the intervention classrooms made greater gains with phonological awareness, vocabulary, and oral narrative retell, but not with letter identification or oral narrative comprehension, compared to the control classrooms^(23)^.

Individuals use mental perceptual representations to recognize information^(24)^, supporting the use of multiple modes of intervention: auditory, motor, and visual. Using motor stimulation for learning spatial terms integrates cognitive processes of language and movement and facilitates the development of a mental representation of the action^(25)^. These findings highlight the need to utilize interactive activities and hands-on games for teaching spatial terms.

### The current investigation

Given the importance of spatial term comprehension and production, and the need for evidence regarding service delivery in the classroom, the current investigation was designed to provide opportunities for learning spatial terms to children in a developmental kindergarten classroom. In the United States, children typically begin kindergarten in August or September at the age of five years, or if they will turn five before December (or earlier, in some school districts) of that year. Developmental kindergarten classes are offered in many school districts for the purpose of providing an extra year of schooling for children who are not emotionally or academically ready to begin kindergarten at the typical kindergarten age. The study aimed to address the following research questions:

1. Does a push-in intervention targeting spatial terms delivered by a speech-language interventionist result in acquisition of spatial terms for children in a developmental kindergarten classroom?
2. Are there differences in the gains in spatial vocabulary comprehension of children with different language ability profiles?

## METHODS

### Participants

Participants were recruited from a rural school district in the United States, in which 62.2% of the children are economically disadvantaged^(26)^. Children in a developmental kindergarten class (n=15) received classroom-based lessons on spatial terms for a period of five weeks. One child in the class was not tested due to absences, but participated in the intervention sessions. Of the 14 students tested, eleven were male, three were female, and all were Caucasian monolingual English speakers. Prior to recruitment, the authors received approval for this study from the Grand Valley State University Research Ethics Committee, called the *Institutional Review Board* (approval number: 17-104-H); the school district superintendent; the building principal; the teacher of the classroom from which children were recruited; and the parents of the children in the classroom, who all signed the Free and Informed Consent Form. All participants provided verbal assent to the testing.

### Procedure

#### Pretest

Prior to the delivery of the language lessons, the children completed the *comprehension of basic concepts* subtest and the four core subtests of the *Comprehensive Assessment of Spoken Language (CASL)* ^(27)^. The *comprehension of basic concepts* subtest asked the children to point to the correct picture out of four options that corresponded to the verbal direction given by the test administrator. The core subtests included *antonyms*, *syntax construction*, *paragraph comprehension of syntax*, *and pragmatic judgment*. The *antonyms* subtest asked the children to name the antonym of specific vocabulary. The *syntax construction* subtest involved providing one word to finish a sentence. *Paragraph comprehension of syntax* entailed answering questions about short stories. *Pragmatic judgment* involved stating what should be done in social situations.

CASL *Core Language Composite* scores were used to group the children into a typically developing (TD) group (n=11), who earned a standard score within one standard deviation of the mean (i.e., greater than 85), and a lower language (LL) group (n=4), who earned a standard score below 85. The cutoff score of 85 was selected as it has been found to accurately differentiate between children with and without language impairment on the CASL^(28)^. This grouping was used in the analysis only, children were not grouped during the lessons and the first author, who delivered the lessons, did not know which children fell into each group. Table 1 depicts the CASL scores for each group and the total sample.

**Table 1:** Scores on the Comprehensive Assessment of Spoken Language (CASL)

	Total		Low CASL Scores		Typical CASL Scores	
Variable	N	M(SD)	N	M(SD)	N	M(SD)
Age (months)	15	66.33(2.41)	4	66.50(2.08)	11	66.27(2.61)
Core SS	15	90.20(17.97)	4	67.50(15.33)	11	98.45(10.04)
Pre BC RS	15	29.73(3.24)	4	27.00(2.94)	11	30.73(2.83)
Pre BC SS	15	97.73(9.07)	4	90.50(5.45)	11	100.36(8.81)
Pre A RS	15	9.40(4.93)	4	4.50(5.80)	11	11.18(3.28)
Pre A SS	15	87.27(15.33)	4	72.25(16.84)	11	92.73(11.00)
Post BC RS	14	30.93(4.81)	4	25.50(4.51)	10	33.10(2.88)
Post BC SS	14	99.07(11.61)	4	86.00(8.67)	10	104.30(7.96)
Post A RS	14	10.43(4.88)	4	6.50(6.66)	10	12.00(3.16)
Post A SS	14	89.43(12.06)	4	81.25(16.46)	10	92.70(8.83)

Caption: RS = raw score; SS = standard score; pre = pre-test; post = post-test; BC = Basic Concepts; A = Antonyms; M = mean; SD = standard deviation

#### Intervention

Half-hour lessons were provided to the developmental kindergarten class in their regular classroom once weekly for five weeks by the first author, a speech-language pathology student who had completed university coursework in language development and disorders. Each lesson taught three to four spatial terms: *over*, *under*, *through*; *before*, *after*, *beginning*, *end*; *above*, *below*, *center*, *around*; *left*, *right*, *front*, and *behind*. Appendix A provides further detail about the lessons. When children learn the relationships between key words and corresponding objects, they have better memorization of the word meanings^(29)^; thus, each lesson began with an introduction of the new words and review of the words targeted previously, presented with printed pictures or illustrations drawn on the board. Next, the children listened to the reading of a book that contained the target words. The reader asked “wh” questions (who, what, when, where, why, how) to allow the students to reflect on what had happened and to predict what would happen next. During the final portion of each session, the children participated in interactive games and activities, as described in Appendix A. During the sixth week of the study, the first author provided a review of the spatial terms covered in the lessons. An original poem, found in Appendix B, was created for this review to include all of the targeted words from the lessons.

#### Posttest

During the week following the intervention, the first author re-administered the *basic concepts* subtest of the CASL, which included the relational terms targeted during the intervention, to assess learning of the terms.

## RESULTS

Data were analyzed using SPSS Statistics. Table 1 displays descriptives for performance on the CASL core composite and the *basic concepts* subtest.

### Pre-post item analysis

For each target word, the percentage of children who did not accurately demonstrate understanding at pre-test but did at post-test was computed for the total group and by language status (TD or LL). See Table 2. A chi-square (χ^2^) test of independence was performed for each target word to assess the relation between language status (TD or LL) and learning that word. There was no association between language status and whether the children who did not know the word at pre-test knew it at post-test.

**Table 2:** Item analysis of learned concept

Word	# Incorrect at pre-test	# (%) Learned	# Incorrect at pre-test TD	# (%) Learned	# Incorrect at pre-test LL	# (%) Learned	χ^2^ π
		(Incorrect at pre-test, correct at post-test)		TD		LL	
First	1	1 (100%)	1	1 (100%)	0	n/a	0.52
Front	4	3 (75%)	2	2 (100%)	2	1 (50%)	0.41
Around	12	5 (42%)	8	4 (50%)	4	1 (25%)	0.14
Last	7	5 (71%)	3	3 (100%)	4	2 (50%)	0.06
Behind	7	5 (71%)	4	4 (100%)	3	1 (33%)	0.54
Center	5	3 (80%)	3	2 (66%)	2	1 (50%)	0.66
Below	6	5 (83%)	3	3 (100%)	3	2 (66%)	0.86
Under	10	8 (80%)	9	6 (67%)	1	2(50%)	0.52
Right	7	9 (69%)	5	0 (0%)	2	2(100%)	0.51
Left	9	2 (22%)	5	2 (20%)	4	0(0%)	0.19

Caption: TD = typically developing; LL = lower language

## DISCUSSION

The current study evaluated the impact of five weekly whole-class language lessons provided to a developmental kindergarten class in a relatively low-income area. These children may be considered at-risk for language deficits, as they are from relatively lower income families and have been identified as not yet ready for kindergarten entry at age five, as evidenced by their placement in developmental kindergarten.

The results of this study suggest that classroom-based language services with activities incorporating visual, verbal, and kinesthetic information delivered by an interventionist with a background in speech-language pathology can be beneficial to students with and without language deficits. Following the intervention, more than half of the children who at pre-test did not demonstrate comprehension of the words *first, front, last, behind, center, below, under,* and *right* did correctly identify pictures representing these words at post-test. *Around* and *left* were the only two of the ten words that were not learned by more than half of the children who did not demonstrate understanding of them at pre-test. For each word, a greater percentage of children with typical language abilities learned the words than the percentage of children with lower language abilities. This was expected, given that children with language deficits need more exposure to new vocabulary in order to learn the words than children with typical language. However, the group differences were not significant, and the children with lower language abilities did demonstrate learning of the new words. This supports the role of the SLP in Tier 1 intervention in RtI or MTSS frameworks. SLPs can provide whole-class learning opportunities using the structure described here, with interactive book-reading followed by interactive games and activities to introduce new concepts.

## CONCLUSION

Future investigations should compare children with low language scores receiving whole-class language lessons to children with low language scores receiving small-group or individual intervention. Additionally, the effectiveness of this intervention should be compared in children of varying socioeconomic status. The children in the current investigation all resided within the same school district catchment area, which is comprised of a relatively low-income population. There may be differences in the effectiveness of the intervention for children in an upper-income area.

## Appendix A Relational Vocabulary Lessons

**Table:**

**Vocabulary**	**Book**	**Activity**
Over, under, through	*We’re Going on a Bear Hunt*	Students demonstrated vocabulary in small groups with pairs forming the barriers to go over, under, and through.
	Michael Rosen	
Beginning, end, before, after	*The Very Hungry Caterpillar*	Students discussed the story after they each placed a food that the caterpillar ate on the board in the order it was consumed. Then they were given directions to line up in a certain order.
	Eric Carle	
Above, below, around, center	*Go Dog, Go!*	Students ran around and below the center of a parachute on the playground. Then they played Duck, Duck, Goose in which two students chased each other around the circle while one student was the goose in the center of the circle.
	P.D. Eastman	
	*Above and Below*	
	Hanako Clulow	
Front, behind, right, left	*The Foot Book*	Students played Simon Says targeting all vocabulary, especially the new target vocabulary. Then they danced to the Hokey Pokey and the Cha Cha Slide to learn left and right.
	Dr. Seuss	
Review	*The Day My Hamster Sammy Escaped!*	Students showed the positional and directional words using cups and erasers. Then they were each given a plastic Easter Egg containing a card with a picture of the vocabulary. Each student attempted to use their word in a sentence while the others listened.
	Maggie Eising	
	*Original poem with moveable characters attached to background on poster	

## Appendix B The Day My Hamster Sammy Escaped!

My hamster Sammy lives in a hamster house

In his cage in the living room right next to the couch

He loves to run and run *around* on his wheel

And skitters across his cage when it’s time for a meal

One day as I opened his cage to feed him…

He popped out of its door *before* I could greet him

And scurried right *through* my hands!

*Before* I could catch Sammy

He ran *behind* me

*Through* the *center* of the living room

He looked so tiny!

In a small crack between the TV and wall

Then he knocked *over* a plant in his frantic scrawl

*Under* the table, then *below* the stairs

He sprinted *around* the dog Dewey who was unaware

That Sammy had escaped!

Then Dewey spotted Sammy and followed after madly

He was *beginning* a chase that was sure to *end* badly

Sammy hurtled straight *through* the door

*Before* Dewey could catch him in one second more

Into the *center* of the kitchen the hamster did scurry

I ran in *after* him in quite a hurry

*Around* the stove and *below* the chairs

*Right* of the sink and *left* of the pairs pears

In *front* of the dog Sammy sprinted in fright

With a jump and a squeak he ran, eyes bright

Dewey swiped his paw but missed by a hair

Then Sammy turned *around after* this scare

Back to the living room

*Around* the couch

*Over* the lampshade

As Dewey crouched

Ready to spring into action when Sammy the hamster went by

But Sammy jumped *over* Dewey who frowned with a sigh

*Below* his outstretched paws Sammy bolted

*Left* of where I stood the hamster jolted

Then stuck in the *center* of Sammy’s paw

The piece of gum I lost is what I saw

Dewey slipped *behind* Sammy and bumped the hamster

Out of the gum trap that once was a disaster!

Sammy ran to the *front* of his cage and *through* its door

No more escaping, not anymore

Next time, *before* I feed Sammy again,

I will be sure to bring my old friend

Dewey the dog

Who chased Sammy back to his home

Because that is where Sammy is meant to roam
